# Centre of pressure progression and gait parameter deviations may be related to second rocker dysfunction in children with flat feet

**DOI:** 10.1186/1757-1146-7-S1-A47

**Published:** 2014-04-08

**Authors:** Alpesh Kothari, Catriona Kerr, Julie Stebbins, Amy B Zavatsky, Tim Theologis

**Affiliations:** 1 Nuffield Department of Orthopaedics Rheumatology and Musculoskeletal Sciences, Nuffield Orthopaedic Centre, Windmill Road, Oxford, OX3 7LD, UK; 2 Department of Engineering Science, University of Oxford, Oxford, OX1 3PJ, UK; 3 Oxford Gait Laboratory, Nuffield Orthopaedic Centre, Oxford OX3 7LD, UK

## Background

The Centre of Pressure Progression (COPP) is thought to be a useful measure of dynamic function of the foot [1]. The COPP has been used as an outcome measure in flat foot surgery, with an improved COPP defining a successful surgical result [2]. It is, however, unclear how the COPP varies in children with flat feet (FF) compared to those with normal arches (NA) and how this relates to dynamic function of the foot. The aim of this study was to quantify the differences in COPP between flat and normal arched children and also assess how these related to temporal-spatial gait parameters.

## Patient/materials and methods

Forty children with NA and twenty-one with FF (age 8-15) underwent dynamic pedobarography with the Novel Emed-M pressure plate system. A representative pressure trial at a self-selected walking speed was masked into three foot regions (heel, midfoot and forefoot). The position of the COPP line with respect to the long axis of the foot was calculated and interpolated to sixty points and this was normalised to foot size. Mean differences between COPP position for FF and NA were calculated with 95% confidence intervals (CI) for each interpolated point. The percentage of roll over process (ROP) in each foot region was calculated and differences between groups were assessed using a t-test. Walking speed normalised (NWS) to leg length was obtained from three dimensional motion analysis.

## Results

**Figure 1:**
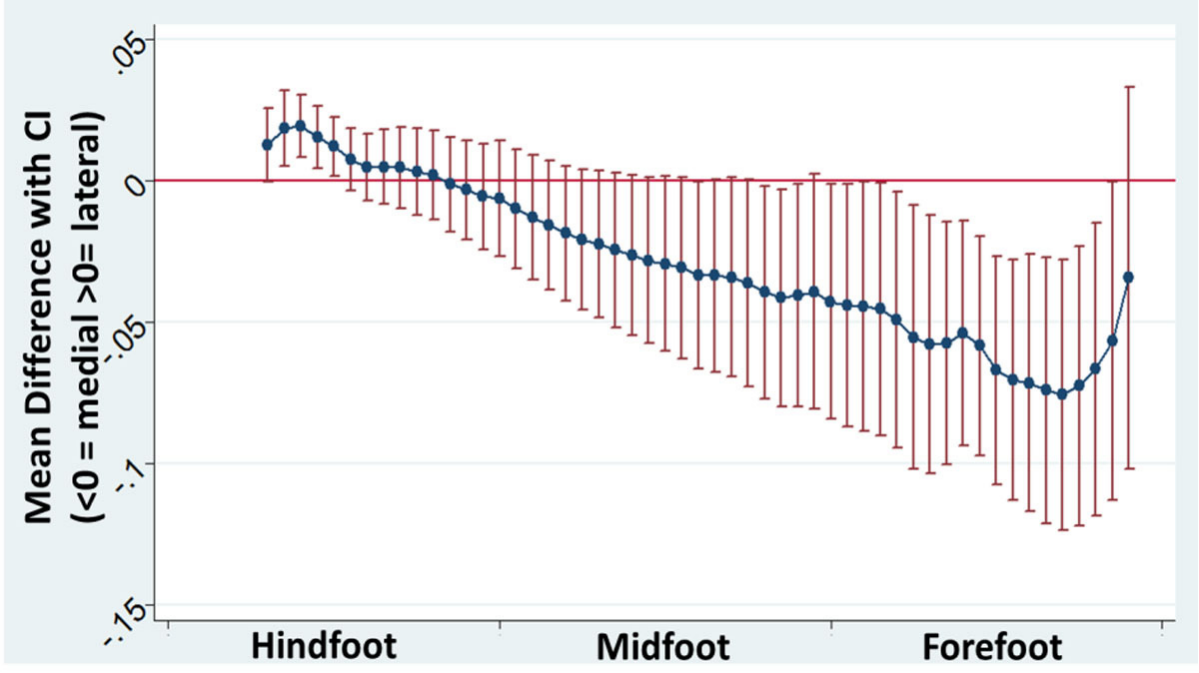
Mean difference and 95% confidence intervals (CIs) of COPP, as a proportion of foot width, between FF and NA groups. Significant differences are noted where confidence intervals do not cross zero.

There were no significant age or gender differences between the FF and NA group. The COP was more laterally placed in the FF group at initial contact, but diverged medially as it progressed to the forefoot (figure 1). The timings of the ROP demonstrated a significantly decreased percentage of the ROP in the forefoot region of the FF group compared to the NA group; 50% vs 55% (p=0.03). NWS was significantly slower in the FF group compared to the NA group (p=<0.001).

## Conclusion

In this study we demonstrate that FF children have altered COPP compared to NA. The biggest difference is a more medial position of COP in the forefoot. This observation is probably related to the hindfoot eversion and forefoot pronation seen in flat footed individuals. This result combined with the reduction in percentage of ROP in the forefoot region and reduced walking speed would suggest a dysfunction in progression of the second rocker.
